# P-393. Treatment Switch among Medicare-insured People with HIV and Gaps in Care

**DOI:** 10.1093/ofid/ofaf695.610

**Published:** 2026-01-11

**Authors:** Suying Li, Mary J Christoph, Uche Mordi, Haifeng Guo, David T Gilbertson, Tianye Cui, Travis Lim, Neia Prata Menezes

**Affiliations:** Chronic Disease Research Group, Minneapolis, Minnesota; Gilead Sciences, Inc., Foster City, California; Gilead Sciences, Inc., Foster City, California; Chronic Disease Research Group, Minneapolis, Minnesota; Chronic Disease Research Group, Minneapolis, Minnesota; Chronic Disease Research Group, Minneapolis, Minnesota; Gilead Sciences, Inc., Foster City, California; Gilead Sciences, Inc., Foster City, California

## Abstract

**Background:**

The purpose of this study was to describe treatment switch by antiretroviral therapy (ART) regimen in People with HIV (PWH) insured by Medicare who have significant gaps in care prior to resuming ART and those with low adherence while on therapy.

**Table 1.**
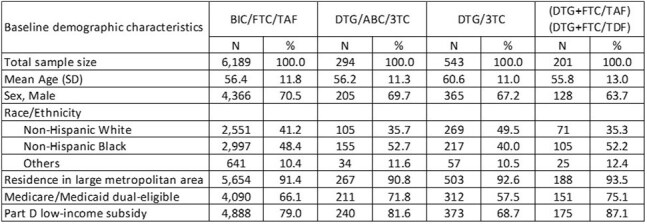
Baseline demographics of Medicare beneficiaries with HIV who have ≥90-day gap prior to initiating index ART

**Table 2.**
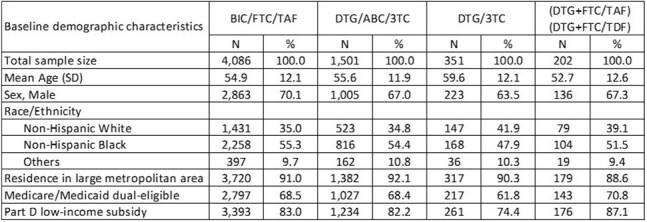
Baseline demographics of Medicare beneficiaries with HIV who have low adherence with PDC < 85% while on the index ART

**Methods:**

This retrospective cohort study extracted data for Treatment-experienced (TE) PWH age 18 or older who initiated ART between 1/1/2018 and 9/30/2022 and were insured by Medicare fee-for-service or Medicare Advantage. Index date was defined as the earliest date of initiating ART with one of the following: BIC/FTC/TAF, DTG/3TC, DTG/ABC/3TC, or DTG+FTC/TAF or DTG+FTC/TDF (MTRs). We examined two study cohorts: (1) TE PWH with ≥ 90-day gap in treatment in the 6 months prior to initiating the index regimen and (2) TE PWH with low adherence on the index therapy measured by proportion of days covered (PDC) < 85% in the first year of follow-up. PWH were followed from index date until switch, censoring at discontinuation, death, end of Medicare enrollment, or 12/31/2022. Switch was defined as starting a new ART regimen within 90 days of the end of the last fill for the index ART. Treatment persistence until switch was evaluated using Kaplan-Meier curves and the log-rank test. Adjusted hazard ratios (HRs [95% CI]) were calculated to assess risk of switch after inverse propensity score treatment weighting.

**Figure 1.**
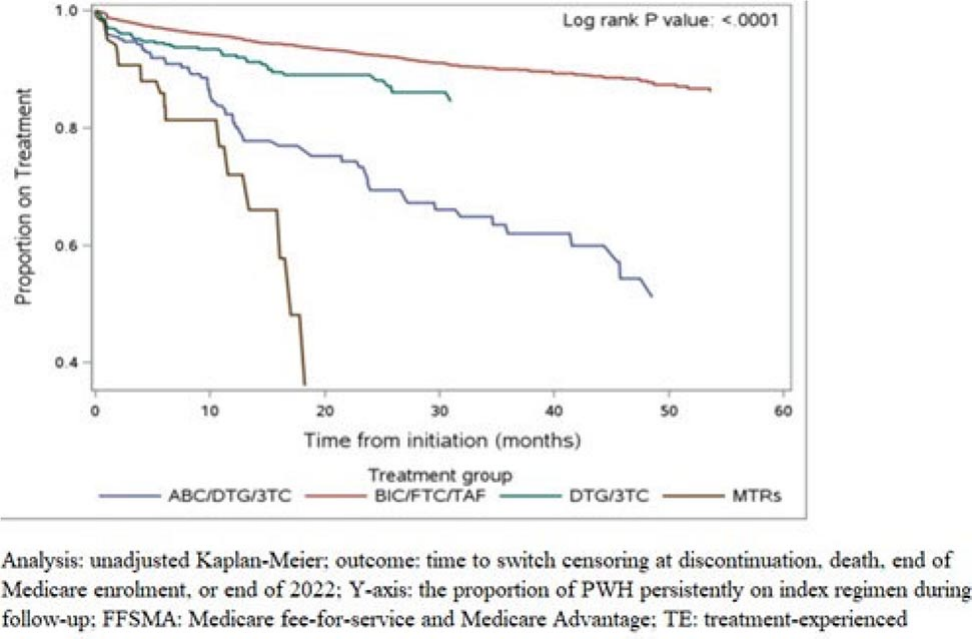
Treatment persistence in Medicare beneficiaries with HIV who have ≥90-day gap prior to initiating ART index regimen

**Figure 2.**
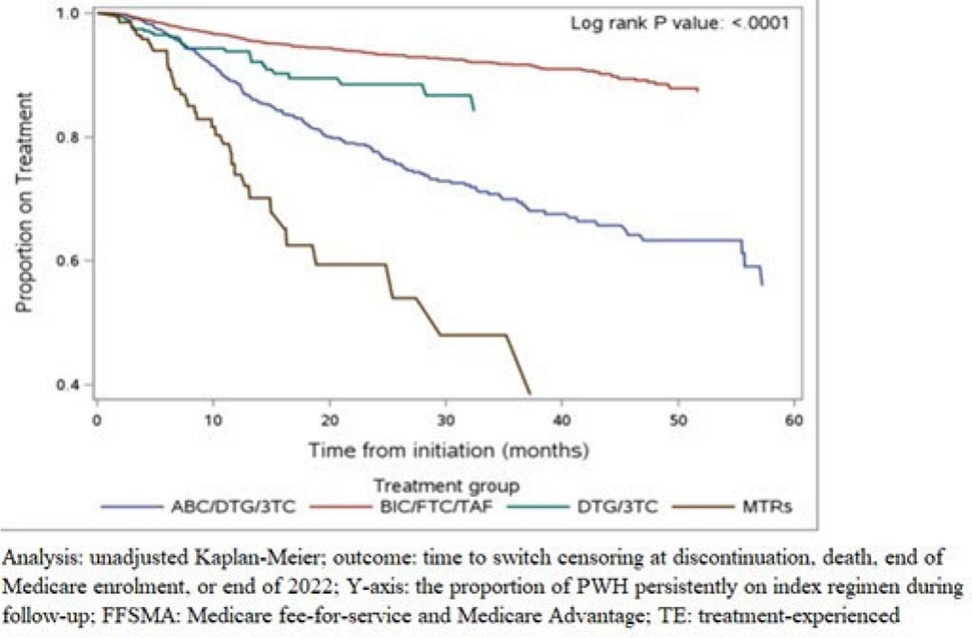
Treatment persistence in Medicare beneficiaries with HIV who have low adherence with PDC < 85% while on the index ART

**Results:**

Among 30,205 TE PWH, 7,227 initiated ART after a gap of ≥ 90 days (Table 1); and 6,140 had a PDC < 85% while on the index regimen (Table 2). Both study cohorts showed differences in treatment switch by ART, with PWH initiating BIC/FTC/TAF having the lowest likelihood of switch compared to other regimens (log-rank test p< 0.0001; Figures 1 & 2). Among PWH with a ≥90-day gap prior to initiating ART, adjusted HRs were 1.65 (1.21, 2.24) for DTG/3TC, 4.05 (3.07, 5.34) for DTG/ABC/3TC, and 7.83 (5.47, 11.22) for MTRs compared to BIC/FTC/TAF. Among the PDC < 85% group, adjusted HRs for switch were 1.65 (1.08, 2.53) for DTG/3TC, 3.45 (2.87, 4.13) for DTG/ABC/3TC, and 7.10 (5.02, 10.04) for MTRs compared to BIC/FTC/TAF.

**Conclusion:**

Among TE PWH who had a gap in care prior to initiating therapy or low adherence to the index therapy, individuals who indexed on BIC/FTC/TAF had lower likelihood of switch versus those starting other regimens.

**Disclosures:**

Suying Li, PhD, Amgen: Grant/Research Support|Gilead Sciences, Inc.: Grant/Research Support|GSK: Grant/Research Support Mary J. Christoph, PhD, MPH, AstraZeneca: Advisor/Consultant|AstraZeneca: Employee|AstraZeneca: Stocks/Bonds (Public Company)|Gilead Sciences, Inc.: Employee|Gilead Sciences, Inc.: Stocks/Bonds (Public Company) Uche Mordi, PharmD, MS, Gilead Sciences, Inc.: Stocks/Bonds (Public Company) Haifeng Guo, MS, Amgen: Grant/Research Support|Gilead Sciences, Inc.: Grant/Research Support|GSK: Grant/Research Support Travis Lim, MSc, DrPH, Gilead Sciences, Inc.: Employee|Gilead Sciences, Inc.: Stocks/Bonds (Public Company) Neia Prata Menezes, PhD, Gilead Sciences, Inc.: Stocks/Bonds (Private Company)

